# Clay Minerals Affect the Solubility of Zn and Other Bivalent Cations in the Digestive Tract of Ruminants In Vitro

**DOI:** 10.3390/ani11030877

**Published:** 2021-03-19

**Authors:** Maria Schlattl, Marzell Buffler, Wilhelm Windisch

**Affiliations:** Chair of Animal Nutrition, TUM School of Life Sciences, Technical University of Munich, Liesel-Beckmann-Str. 2, 85354 Freising, Germany; marzell.buffler@googlemail.com (M.B.); wilhelm.windisch@tum.de (W.W.)

**Keywords:** clay, zinc, trace metals, rumen, abomasum, duodenum, solubility, in vitro

## Abstract

**Simple Summary:**

Adverse weather conditions and harvesting technique have broad effects on forage quality including contamination with soil particles, e.g., clay minerals. Clay minerals are organised in a layered structure which enables adsorption of bivalent cations. Accordingly, ingested clay minerals may interact with dietary bivalent trace elements, such as Cu, Fe, Mn, and Zn. This study aimed to assess the relationship between clay mineral ingestion and the solubility of dietary trace elements along the digestive tract in vitro. In the presence of clay minerals, we found a reduction of solubilised Zn, Cu, and Mn under ruminal, abomasal, and duodenal conditions. However, clay minerals led to an increase in dissolved Fe under abomasal and duodenal conditions. Therefore, ingested clay minerals may be assumed to alter the solubility of essential dietary trace elements in the digestive tract of ruminants.

**Abstract:**

Ruminants ingest large quantities of clay minerals along with inorganic soil constituents in roughages. The layered structure of clay minerals, however, may adsorb cations and may, thus, interfere with the ruminants’ supply of essential trace metals, such as Zn, Mn, Cu, and Fe. As quantitative knowledge about interactions between clay ingestion and essential trace metal metabolism are largely lacking, this in vitro study focussed on the effect of clay on the solubility of dietary Zn and other bivalent trace metals in the digestive tract of ruminants. Therefore, buffered rumen fluid was used for the simulation of ruminal conditions (RC), acidified rumen fluid (pH 2) was used for abomasal conditions (AC), and duodenal chyme was used for duodenal conditions (DC). These media were added with gradient levels of zinc and incubated at 39 °C for 24 h in the absence or presence of clay minerals. Soluble Zn, Cu, Mn, and Fe were derived by centrifugation (10,000× *g*) of incubated media, and the supernatants were analysed. Clay depressed the solubility of added Zn in ruminal (65.3% vs. 16.5%), abomasal (97.7% vs. 33.7%), and duodenal conditions (41.3% vs. 21.1%), the results of which were statistically significant (*p* < 0.001). Moreover, clay reduced dissolved Cu (µg/mL) (RC: 0.13 vs. 0.10; AC: 0.16 vs. 0.13; DC: 0.10 vs. 0.08) and Mn (µg/mL) (RC: 3.00 vs. 1.80; AC: 5.53 vs. 4.80; DC: 3.18 vs. 1.77) (*p* < 0.05 in all cases). The presence of clay minerals increased the concentrations of solubilised Fe (µg/mL) in abomasal (1.80 vs. 2.86, *p* < 0.05) and duodenal conditions (1.76 vs. 2.67; *p* < 0.05). In total, the present in vitro study demonstrates the potential of clay minerals ingested with ruminant feeds for depressing the solubility of dietary Zn, as well as the depression of dietary Cu and Mn along the passage of the digesta from the rumen until the duodenum. Additionally, clay minerals may release Fe into the digesta.

## 1. Introduction

Soil contamination affects forage quality. The crude ash content of forages is an indicator of undesired contamination with inorganic soil components and should not exceed 10% of dry matter (DM) [1]. In practice, crude ash concentrations (e.g., grass silages) may reach up to 30% of DM due to problems with weather conditions and harvesting technology [2,3,4]. Hence, ruminants may ingest large quantities of soil contaminants [5,6,7].

Most of the inorganic fraction of soil constitutes silicates in conjunction with cations [8]. In general, silicates are known as indigestible feed contaminants that cannot be absorbed in the digestive tract, but they may interact with digestive processes. One main category of silicates is represented by clay minerals. The composition of clay minerals and their potential to interfere with digestion depend on their origin and vary widely with geographical location [8]. In general, clay minerals are organised into a layered structure of silica and alumina sheets, which has a surface with negative charges that may adsorb cations, such as protons and metal ions [9,10]. The group of 1:1 clay minerals includes equal counts of octahedral and tetrahedral sheets (e.g., the dioctahedral kaolinite), whereas 2:1 clay minerals exhibit a sandwich structure of one octahedral sheet between two opposing tetrahedral sheets (e.g., smectites, illites). Cation sorption sites occur, where the resulting layered structure of octahedral and tetrahedral sheets becomes negatively charged [11]. Thus, the pattern of clay minerals present in the soil substantially influences the overall adsorptive potential, with the type of clay minerals and the density of the layer structures within the clay mineral being the most prominent determinants.

Antoniadis et al. [12] showed that the adsorption of any cation generally depends on the affinity of the soil surface for a given element. Cations with a higher atomic weight, a higher ionic and smaller hydrated radius, and a greater hydrolysis constant are preferably adsorbed by other elements [13]. In general, the sorption sites of clay minerals can be categorised into specific and nonspecific sites [14]. At higher cation concentrations in the liquid surrounding the clay, specific sorption sites become increasingly occupied or even blocked due to overlaps. Therefore, adsorption becomes more and more nonspecific and adsorption capacities become exhausted at high cation concentrations [15,16,17].

The adsorption characteristics of clay minerals have been well described in conditions found in soils. Interestingly, clay minerals may adsorb trace minerals with high relevance to animal nutrition, such as Cd, Cu, Pb, and Zn [18,19,20]. Chantawong et al. [19] studied two different types of clay minerals and found higher adsorption capacities for Zn than for Cu, Cd, and Pb. These findings agree with the observations of Veli and Alyüz [21], who found that higher adsorption capacities exist for Zn compared to Cu. The capacity of clay to adsorb trace minerals also depends on the ambient pH, and a pH level less than 6 causes excessive protonation of the negatively charges sorption sites [18,19,22]. In addition, the susceptibility of trace minerals themselves to be adsorbed by clay varies with the ambient pH due to solubility (high solubility at low pH; pronounced formation of insoluble hydroxides above a pH of 6) [20,23].

In total, ambient pH values of ~4–6 represent the sensible range of adsorptive interactions between trace minerals and clay. This range is fully covered by the pH in the contents of the digestive tract of ruminants and switches from mildly acidic to overly acidic to almost neutral along the passage through the respective compartments (rumen: 5.5 to 6.9; abomasum: around 2; duodenum: 2.5 to 6.9 [24]). Accordingly, the presence of clay in feeds might induce the processes of trace mineral adsorption (rumen), release through desorption (abomasum), and adsorption again (duodenum); hence, it might affect the supply status of rumen microorganisms, as well as of the host, with essential trace minerals. Since, to our knowledge, these aspects have not yet been investigated in detail, the present study focussed on the in vitro adsorptive potential of clay minerals under ruminal, abomasal, and duodenal conditions for bivalent trace elements, including Zn in particular.

## 2. Materials and Methods

Clay minerals were obtained from Stephan Schmidt KG, Dornburg-Langendernbach, Germany. The applied mixture (seen in Table 1) was composed of 90% bentonite (primarily 2:1 clay minerals) and 10% kaolinite (1:1 clay mineral) and illite (2:1 clay mineral).

After digestion with nitric acid (microwave digestion: 0.7 g clay minerals digested with 6 mL of nitric acid and 3 mL of hydrogen peroxide), the clay mineral mixture was analysed for its mineral content (Table 2) using inductively coupled plasma mass spectrometry (ICP-MS; PerkinElmer, Waltham, MA, USA). Indium and germanium were used as internal standards for the control and correction of fluctuations. Calibration was performed using commercial standard solutions. Each sample was analysed at least twice in order to keep the coefficients of variation below 10%. Minerals not solubilised by nitric acid digestion were considered as indigestible and, therefore, irrelevant for further calculations.

The clay was incubated in two levels (clay vs. control) in different media simulating the conditions of the rumen, the abomasum, and the duodenum content. The incubation procedures followed the Hohenheim feed value test of Menke and Steingass [25]. Diverging from their method, gas production was not recorded, and the following incubation media were used:

(a) Simulation of ruminal conditions

Rumen fluid was obtained before morning feeding from a nonlactating dairy cow equipped with a rumen fistula and fed 50:50 grass hay/corn silage and 50 g/day of a commercial mineral mix containing 8.000 mg zinc oxide/kg. The rumen fluid was immediately filtered through two layers of cheese cloth into a vessel without air access and transported to the laboratory at a temperature of 39 °C. The constantly tempered rumen fluid was added to a reduced buffer solution, as described in detail by Menke and Steingass [25]. This mixture underwent treatment with CO_2_ in a water bath at 39 °C and was stirred using a magnetic stirrer. For the clay treatment, 200 mg of clay mineral mixture and 28 mL of incubation media were added to prewarmed syringes. Syringes for the control group were filled without the addition of clay minerals. Each syringe was then supplemented with 2 mL of a nitrous zinc standard solution, containing 0 (double-distilled water), 20, 40, 60, and 80 µg/mL Zn in order to generate graded levels of Zn concentrations in the medium (0, 1.3, 2.7, 4.0, and 5.3 µg/mL). Subsequently, syringes were placed in a rotating apparatus (1 rpm) inside a drying oven, which was adjusted to a constant temperature of 39 °C. Samples were incubated for exactly 24 h and centrifuged afterward at 10,000× *g* for 15 min. The supernatant was filtrated (ashless filters, Grade 389, Ahlstrom Munkjö, Helsinki) and stored at −18 °C until further analysis.

(b) Simulation of abomasal conditions

Simulation of abomasal conditions was conducted in the same way as described above for rumen conditions except the solutions were acidified to a pH of 2.0 with hydrochloric acid (5 mol/L) prior to 24 h incubation.

(c) Simulation of duodenal conditions

Duodenal conditions followed the procedure for ruminal conditions, except for the use of duodenal chyme instead of rumen fluid. The duodenal chyme was obtained from the same animal via a fistula at the proximal duodenum prior to morning feeding.

Each incubation medium (ruminal, abomasal, duodenal) comprised a total of 10 treatment combinations (2 clay categories × 5 levels of added Zn). Each combination was replicated threefold, resulting in 30 syringes per incubation medium. The entire experimental setup was repeated one more time using new rumen fluid and duodenal chyme. Therefore, each combination (media × clay category × levels of added Zn) was replicated sixfold in total.

### 2.1. Chemical Analysis

Frozen supernatants were thawed at room temperature, and 2 mL of each was subsequently mineralised through microwave digestion (CEM GmbH, Kamp-Lintfort, Germany) using 2.5 mL of nitric acid and 1.5 mL of hydrogen peroxide. Digested samples were analysed for Zn, Cu, Fe, and Mn by ICP-MS as described above for clay minerals.

### 2.2. Statistical Analysis

Mineral concentrations in the supernatants of each incubation media without Zn addition were submitted for an analysis of variance (ANOVA) considering the fixed factors of *clay* (two levels) and *experimental replicate* (two levels). Means of the clay levels (clay vs. control) were tested for statistical difference using the F-test of the factor *clay* in the ANOVA, with *p* < 0.05 considered statistically significant.

In order to evaluate the response of soluble Zn to the absence/presence of clay, a multiple regression analysis was performed comparing the quantities of Zn (in mg) found in the liquid phase of each syringe (solubilised Zn) with the total amounts of Zn (in mg) added to the system. The analysis was performed separately for each incubation media using a mixed statistical model (PROC MIXED; SAS 9.4) comprising two intercepts and two linear and quadratic slopes each for the control and clay variant and considering repeated measurements (*n* = 3) per level of Zn addition (*n* = 5) and clay variant (*n* = 2). In the case of nonsignificant (*p* > 0.05) quadratic effects, only linear models were calculated. The intercepts and slopes were compared pairwise using a *t*-test for statistical difference at *p* < 0.05.

## 3. Results

### 3.1. Effect of Clay Minerals on the Concentrations of Dissolved Trace Elements under Ruminal, Abomasal, and Duodenal Conditions

Table 3 illustrates the pH of the media at the end of incubation and the mean concentrations of dissolved Zn, Cu, Mn, and Fe for clay and control under ruminal, abomasal, and duodenal conditions without the addition of Zn.

On average, the pH was approximately 7.02 for rumen fluid, 2.0 for the acidified rumen fluid used for simulation of abomasal conditions, and 3.58 for duodenal chyme taken at the proximal duodenum. In rumen fluid, without added clay, the concentrations of soluble Zn, Cu, Mn, and Fe were 0.095, 0.13, 3.00, and 1.64 µg/mL, respectively. Addition of clay reduced the concentrations of dissolved Zn, Cu, and Mn by 42%, 23%, and 40%, while Fe concentrations remained unchanged. Under abomasal conditions, clay depressed soluble Zn, Cu, and Mn by 49%, 18%, and 13% compared to the control level 0.196, 0.16, and 5.53 µg/mL), respectively, while soluble Fe almost doubled from 1.80 to 2.86 µg/mL. Similar reactions were observed under duodenal conditions. Clay reduced soluble Zn, Cu, and Mn from 0.20, 0.10, and 3.18 µg/mL by 52%, 20%, and 44%, respectively, while soluble Fe increased from 1.76 µg/mL by 52%.

### 3.2. Solubility of Supplemental Zinc under Ruminal, Abomasal, and Duodenal Conditions

**Figure 1:**
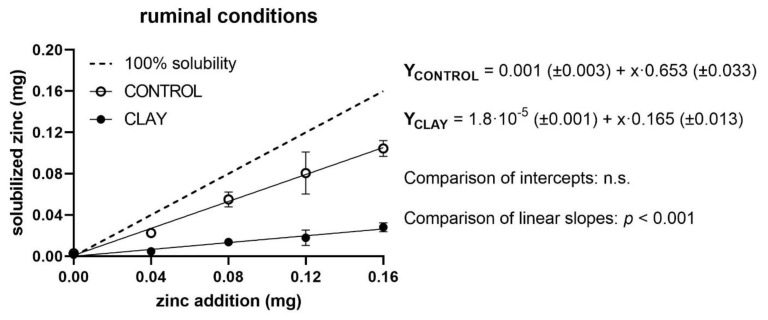
Solubilised zinc (mg) under ruminal conditions with (clay) and without (control) clay mineral addition and supplementation of different zinc doses (mg). The dashed line shows the curve progression at 100% solubility of the added zinc. The *p*-values display the significance of differences between the intercepts and slopes of the regression line of clay and control (n.s. = not significant).

**Figure 2:**
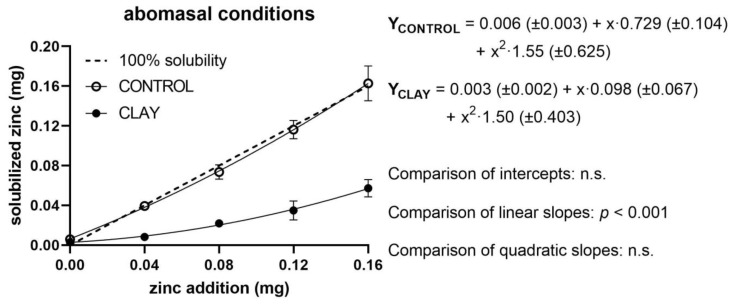
Solubilised zinc (mg) under abomasal conditions with (clay) and without (control) clay mineral addition and supplementation of different zinc doses (mg). The dashed line shows the curve progression at 100% solubility of the added zinc. The *p*-values display the significance of differences between the intercepts and slopes of the regression line of clay and control (n.s. = not significant).

**Figure 3:**
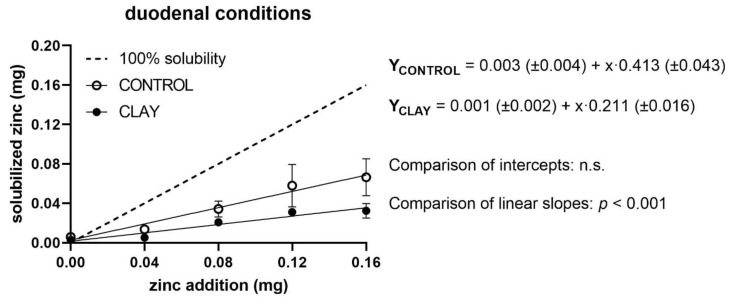
Solubilised zinc (mg) under duodenal conditions with (clay) and without (control) clay mineral addition and supplementation of different zinc doses (mg). The dashed line shows the curve progression at 100% solubility of the added zinc. The *p*-values display the significance of differences between the intercepts and slopes of the regression line of clay and control (n.s. = not significant).

The measured levels of quantities of soluble Zn in relation to added Zn, as well as regression lines of both treatment groups (clay and control) and the reference line for 100% solubility, are shown in Figure 1, Figure 2 and Figure 3 for ruminal, abomasal, and duodenal conditions, respectively. For ruminal conditions, regression analyses showed no significant effect of a quadratic term; thus, a linear model was assumed (Figure 1).

The soluble Zn of the control group responded to added Zn in a straight linear fashion with an intercept of virtually zero and a slope of 0.65 mg/mg (equivalent to a solubility of 65%). Added clay did not affect the intercept but reduced the slope to 0.16 mg/mg (equivalent to a solubility of 16%). Again, the relationship exhibited a straight linear characteristic. The depression of the clay slope compared to the control slope was statistically and highly significant (*p* < 0.001).

In the case of abomasal fluid (Figure 2), regression analysis revealed the presence of a statistically significant quadratic effect. The deviation from linearity, however, was quantitatively marginal in both treatments (y_CONTROL_ = 0.001(±0.003) + x·0.977(±0.032); y_CLAY_ = -0.002(±0.002) + x·0.338(±0.023)). Added clay did not affect the intercepts but depressed Zn solubility by 64% compared to control. This was evident when plotting the five differences between the means of control and clay (*y*-axis) against Zn additions (*x*-axis). The respective regression analysis revealed a straight linear function (y_(CONTROL − CLAY)_ = 0.003 ± 0.002 (*p* = 0.13) + x·0.640 ± 0.017 (*p* < 0.0001); *R*^2^ = 0.998) indicating that the depressive effect of clay was constant over the entire range of Zn additions.

Under duodenal conditions, soluble Zn increased with added Zn in a straight linear fashion for both the control and the clay situations (Figure 3). Intercepts of both regression equations were approximately zero and did not differ from each other, while the slopes were significantly depressed by clay (*p* < 0.0001). Accordingly, Zn solubility dropped from 41% to 21% in the presence of clay.

## 4. Discussion

This study aimed to evaluate the binding properties of clay minerals on Zn in the digestive tract of ruminants, since, to our knowledge, the scientific information about this aspect is very limited. Therefore, we conducted an in vitro pilot study using the Hohenheim feed value test as a basis for simulating the digestive situation in the rumen, abomasum, and the duodenum.

First, 200 mg of clay mineral mixture was incubated in 30 mL of rumen fluid for simulating ruminal conditions. Presuming a volume of 100 L of fluid in a rumen and 18 kg of dry matter intake (DMI) per day, our model displays around 0.7 kg clay minerals in the entire rumen or 4% clay of total dietary DM. In practice, crude ash concentration in forages varies ranges from 4% to >20% [2,3,4]. Assuming that clay minerals dominate the soil mineral fraction, which itself dominates the crude ash fraction of feedstuffs [26], our in vitro model seems to represent the practical concentration of clay minerals in forage. Regarding Zn, the amounts added to the in vitro system (0, 0.04, 0.08, 0.12, or 0.16 mg) were equivalent to additional Zn supplies between 0 and around 30 mg per kg of DM (assuming 100 L of rumen fluid and 18 kg DMI/day respectively). Considering that the native Zn contents of grass silages are in the range of 25 to 55 mg/kg DM [27], our in vitro model covers levels of total dietary Zn between around 40 to 70 mg/kg of feed dry matter, which is around the range of recommendations for dairy cows (50 mg Zn/kg feed DM [28]).

The clay used in this study showed high native contents of trace elements such as Zn, Cu, Mn, and Fe, which might have been released into the incubation media. In the cases of Zn, Cu, and Mn, however, the addition of clay even reduced the concentrations of soluble Cu, Mn, and Zn in the ruminal, abomasal, and duodenal incubation media. Apparently, the trace minerals introduced into the test system via the added clay remained tightly bound; in addition, the clay was still capable of binding even more of these trace minerals. In contrast, clay Fe seemed to remain largely bound to its carrier under ruminal conditions, while it was released in the abomasal and duodenal simulations. Sheta et al. [29] showed that certain clay minerals have a high binding potential for Fe, but the extractability of adsorbed Fe was also high. Accordingly, low Fe concentrations in digestive fluids may, in principle, cause a high gradient of Fe concentration and, hence, favour the release of clay Fe into the surrounding media. However, as the adsorptive capacity of clay minerals dropped from neutral toward acidic conditions [19,23], the release of clay Fe became significant when changing the in vitro system from ruminal to abomasal conditions through a reduction in pH (from pH 7.0 to pH 2.0). Interestingly, Fe released from clay remained high under duodenal conditions despite the increase in pH compared to abomasal conditions (pH 2.0 to 3.6). The explanation for this specific behaviour of duodenal fluid remains unclear and requires further investigation. Nevertheless, the clay Fe released into the duodenum might negatively affect the bioavailability of Zn, Cu, and Mn [30,31,32].

In our in vitro system, concentrations of solubilised Zn in the absence of Zn addition were very low (between 0.1 and 0.2 µg/mL), regardless of the simulated conditions. Similar in vivo observations were reported by Spears et al. [33] and Genther and Hansen [34], who measured around 0.3 µg Zn/mL and 0.5 µg Zn/mL in the rumen fluid several hours post feeding. This indicates the rapid removal of solubilised feed Zn into other compartments, such as ruminal microbes and, along with the passage of the digesta through the digestive tract, into mucosal cells. As no mucosa was present in our in vitro system, the low level of solubilised Zn seems to reflect mainly an incorporation of Zn into microbes, which are known for comparably high Zn concentrations. For example, Kennedy et al. [35] reported almost five times higher Zn concentrations in the DM of rumen microbes compared to feed. It may be assumed that these amounts of microbial Zn serve as a quantitatively relevant Zn source for digestive processes behind the abomasum.

In the absence of Zn addition to the in vitro system, the addition of clay further reduced the concentrations of solubilised Zn regardless of the simulated situation. Presumably, the clay competed with the ruminal microflora for soluble Zn. This seemed to also apply to the rumen simulation system at gradients of Zn additions, since the solubility of extra Zn dropped from 65% to 16% when clay was added. These figures suggest a considerable adsorptive potential of clay under ruminal conditions. Presumably, the adsorptive capacity of clay was not exhausted since the depressive effect on Zn solubility responded in a linear fashion up to the highest Zn level. As an alternative hypothesis, added Zn might have been sequestrated by incorporation into rumen microbes. Such an effect, however, would indicate an insufficient Zn supply to the rumen microbiome. This seems to be somewhat unlikely, as overall Zn supply may be considered to be sufficient (see above). Nevertheless, future investigations need to differentiate the localisation of sequestered Zn between the fraction of clay and that of microbial matter. In total, it may be hypothesised that amounts of Zn adsorbed to clay minerals are largely unavailable for other compartments of the rumen fluid, particularly rumen microbes. To our knowledge, the impact of Zn deprivation of rumen microbes through clay intake via feed upon microbial activities is still unknown.

Simulation of abomasal conditions was conducted by acidifying the rumen fluid to pH 2 through the addition of hydrochloric acid, while all other treatments were done in the same way as in the rumen simulation. In the absence of clay, the supplemental zinc was found virtually completely in the soluble phase after 24 h of incubation. This was to be expected since solubility of added nitrous Zn, as well as other inorganic and organic Zn formulations, is known to be very high under such low pH conditions. That applies even to very strong complexing agents such as phytic acid [36]. Added clay, however, reduced solubility by 64%. Indeed, the depressive effect of clay on Zn solubility was much less pronounced compared to the ruminal situation (34% vs. 16% Zn solubility). This may be explained by the rising saturation of the negatively charged surface of clay with protons, along with the lower pH of the incubation media [37]. Nevertheless, clay seems to be capable of adsorbing considerable amounts of Zn even under very low pH conditions.

Duodenal conditions were simulated with duodenal fluid, taken before morning feeding. Processing and treatments were the same as for the ruminal and abomasal simulation. In the absence of clay, Zn solubility was at 40%. This was much lower than with abomasal conditions, which may be explained in part by higher pH values (pH 3.6 vs. 2.0). However, solubility remained also considerably below ruminal conditions despite lower pH (pH 3.6 vs. 7.0). Similar to ruminal and abomasal conditions, clay also depressed Zn solubility in the duodenal fluid, but the extent of this depression was less pronounced than under ruminal conditions (21% vs. 16%). This may be explained by the type of binding sites of clay minerals for cations, which are both specific and nonspecific [14]. The latter might be saturated to a higher extent under duodenal conditions since the DM content of the chyme is higher [24] and, hence, presumably also the overall density of cations. Nonetheless, clay was also able to bind considerable amounts of Zn under duodenal conditions.

## 5. Conclusions

The clay tested in our study showed a significant potential to adsorb Zn, Mn, and Cu under ruminal, abomasal, or duodenal conditions. The respective quantities of trace minerals may be assumed to be largely withdrawn from utilisation by intestinal microbes and finally from absorption through transport proteins of intestinal mucosa. Furthermore, the clay released Fe into the media, which might provide further pressure on the absorbability of Zn, Mn, and Cu [30,31,32]. In addition, the adsorptive potential of clay could not be saturated within the tested range of added Zn, which further highlights the putative potential of clay to negatively affect the intestinal availability of Zn. In vivo studies, however, are still lacking. Future studies need to verify the observed in vitro effects under practical feeding conditions, particularly to characterise the potential of clay to depress Zn bioavailability depending on its composition of clay minerals.

## Figures and Tables

**Table 1:** Characteristics of the clay mineral mixture.

Main Components	90% bentonite (smectite, quartz) 10% kaolinite, illite	
Chemical Composition	60.9% SiO_2_, 17.5% Al_2_O_3_, 11.9% Fe_2_O_3_, 3.32% MgO, 2.84% TiO_2_, 2.35% CaO, 0.99% K_2_O, 0.20% Na_2_O	
Cation Exchange Capacity	77.6 meq/100 g	
Particle Size (vol. Weighted Mean)	33.2 µm	

Data on the clay mineral characteristics were provided by the supplier.

**Table 2:** Analysed mineral content of the clay mineral mixture after digestion with nitric acid.

Ca	Mg	Na	Fe	Mn	Zn	Cu
(g/kg DM)				(mg/kg DM)		
12.5	16.1	0.497	51.7	829	64.1	24.9

**Table 3:** Mean concentration (µg/mL) of dissolved trace elements in samples supplemented with (clay) and without (control) clay minerals under ruminal, abomasal, and duodenal conditions after 24 h incubation and initial pH value of digestive fluids.

Parameter	Treatment	Ruminal Conditions		Abomasal Conditions		Duodenal Conditions	
pH		7.02 ± 0.11		2.00 ± 0.00		3.58 ± 0.24	
		Mean	SEM	Mean	SEM	Mean	SEM
Zn (µg/mL)	Control	0.095 ^a^	0.004	0.196 ^a^	0.019	0.200 ^a^	0.025
	Clay	0.055 ^b^	0.011	0.099 ^b^	0.018	0.095 ^b^	0.003
Cu (µg/mL)	Control	0.13 ^a^	0.002	0.16 ^a^	0.004	0.10 ^a^	0.005
	Clay	0.10 ^b^	0.003	0.13 ^b^	0.003	0.08 ^b^	0.002
Mn (µg/mL)	Control	3.00 ^a^	0.022	5.53 ^a^	0.04	3.18 ^a^	0.098
	Clay	1.80 ^b^	0.082	4.80 ^b^	0.148	1.77 ^b^	0.065
Fe (µg/mL)	Control	1.64 ^a^	0.128	1.80 ^b^	0.150	1.76 ^b^	0.079
	Clay	1.81 ^a^	0.169	2.86 ^a^	0.130	2.67 ^a^	0.159

SEM = standard error of the mean. ^a,b^ Superscript letters indicate statistically significant differences between control and clay within each trace mineral and medium (*p* < 0.05).

## Data Availability

The data presented in this study are available on request from the corresponding author.
